# Western Diet Causes Obesity-Induced Nonalcoholic Fatty Liver Disease Development by Differentially Compromising the Autophagic Response

**DOI:** 10.3390/antiox9100995

**Published:** 2020-10-15

**Authors:** Ines C. M. Simoes, Agnieszka Karkucinska-Wieckowska, Justyna Janikiewicz, Sylwia Szymanska, Maciej Pronicki, Pawel Dobrzyn, Michal Dabrowski, Agnieszka Dobrzyn, Paulo J. Oliveira, Hans Zischka, Yaiza Potes, Mariusz R. Wieckowski

**Affiliations:** 1 Nencki Institute of Experimental Biology of Polish Academy of Sciences, 02-093 Warsaw, Poland; i.simoes@nencki.edu.pl (I.C.M.S.); j.janikiewicz@nencki.edu.pl (J.J.); p.dobrzyn@nencki.edu.pl (P.D.); m.dabrowski@nencki.edu.pl (M.D.); a.dobrzyn@nencki.edu.pl (A.D.); 2 Department of Pathology, The Children’s Memorial Health Institute, 04-730 Warsaw, Poland; a.karkucinska-wieckowska@ipczd.pl (A.K.-W.); s.szymanska@ipczd.pl (S.S.); m.pronicki@ipczd.pl (M.P.); 3 CNC-Center for Neuroscience and Cell Biology, UC-Biotech, University of Coimbra, Biocant Park, 3060-197 Cantanhede, Portugal; pauloliv@cnc.uc.pt; 4 Institute of Toxicology and Environmental Hygiene, School of Medicine, Technical University Munich, D-80802 Munich, Germany; zischka@helmholtz-muenchen.de; 5 Institute of Molecular Toxicology and Pharmacology, Helmholtz Center Munich, German Research Center for Environmental Health, D-85764 Neuherberg, Germany

**Keywords:** mitochondria, oxidative stress, peroxisomes, autophagy, steatosis

## Abstract

Nonalcoholic fatty liver disease (NAFLD) is characterized by the development of steatosis, which can ultimately compromise liver function. Mitochondria are key players in obesity-induced metabolic disorders; however, the distinct role of hypercaloric diet constituents in hepatic cellular oxidative stress and metabolism is unknown. Male mice were fed either a high-fat (HF) diet, a high-sucrose (HS) diet or a combined HF plus HS (HFHS) diet for 16 weeks. This study shows that hypercaloric diets caused steatosis; however, the HFHS diet induced severe fibrotic phenotype. At the mitochondrial level, lipidomic analysis showed an increased cardiolipin content for all tested diets. Despite this, no alterations were found in the coupling efficiency of oxidative phosphorylation and neither in mitochondrial fatty acid oxidation (FAO). Consistent with unchanged mitochondrial function, no alterations in mitochondrial-induced reactive oxygen species (ROS) and antioxidant capacity were found. In contrast, the HF and HS diets caused lipid peroxidation and provoked altered antioxidant enzyme levels/activities in liver tissue. Our work provides evidence that hepatic oxidative damage may be caused by augmented levels of peroxisomes and consequently higher peroxisomal FAO-induced ROS in the early NAFLD stage. Hepatic damage is also associated with autophagic flux impairment, which was demonstrated to be diet-type dependent. The HS diet induced a reduction in autophagosomal formation, while the HF diet reduced levels of cathepsins. The accumulation of damaged organelles could instigate hepatocyte injuries and NAFLD progression.

## 1. Introduction

Nonalcoholic fatty liver disease (NAFLD) is the leading cause of chronic liver disease worldwide, affecting 25% of the population [1]. NAFLD comprises steatosis (nonalcoholic fatty liver (NAFL)), nonalcoholic steatohepatitis (NASH), cirrhosis and hepatocarcinoma [2]. NAFLD is highly correlated with the hallmark features of metabolic syndromes, such as obesity and type 2 diabetes, which can arise because of dietary habits. Hepatic adaptations to maintain metabolic homeostasis are fine-tuned by cellular bioenergetics, lipid metabolism and quality control mechanisms [3]. However, the effect of diet composition on the metabolic rearrangement underlying NAFLD remains incompletely elucidated.

Accumulating evidence has shown that the consumption of hypercaloric diets is a key factor in the onset of NAFL [4]. Saturated fatty acids (SFAs)-enriched diets induce a phenotype in rodent models that closely resembles the metabolic profile of the human disease with body weight gain, insulin resistance (IR), impaired lipid metabolism and liver steatosis after 10 weeks of feeding [5,6,7]. In some patients, NAFL is also strongly associated with carbohydrate intake. Fructose per se has been reported to induce hepatic steatosis in rodents and humans [8,9]. Only a few studies have described the effect of a high-sucrose (HS) diet on NAFLD progression. When combined with a high-fat (HF) diet, sucrose is linked to the development of NASH with fibrosis [10]. In vivo studies have shown that a high-fat, high-sucrose (HFHS) diet, resembling unhealthy Western eating habits, causes most of the features of human NASH, but the pathognomonic mechanisms underlying disease development are still unclear [11].

Mitochondria increase fatty acids (FAs) β-oxidation (mt-FAO) to restrain hepatic fat accumulation [12]. However, the abolished mt-FAO response in NAFLD can be associated with different mitochondrial alterations, such as reduced oxidative phosphorylation (OXPHOS), diminished ATP production and enhanced sensitivity for mitochondrial permeability transition pore (mPTP) opening [12,13,14]. The progression of NAFL towards NASH in a nonreversible manner was reported to involve a prooxidative state and mitochondrial-induced reactive oxygen species (ROS) production, which ultimately alter cellular signaling cascades leading to hepatocellular inflammation and fibrosis [15,16,17]. Conversely, recent evidence has questioned the role of oxidative stress as a trigger of NAFL progression. Einer et al. showed structural and functional mitochondrial alterations with no signs of oxidative stress in a NAFLD mouse model [14]. While previous NAFLD studies mainly focused on the combined effect of fat and sucrose, the individual effect of each nutrient in metabolic cellular pathways has not been compared.

To determine the role of HF, HS and HFHS diets in the development of NAFL in mice, we examined hepatic redox- and mitochondrial-related alterations. In this study, we demonstrate that the different diets have distinct effects on NAFL. Our results proved that sucrose is the crucial nutrient for promoting de novo lipogenesis (DNL), whereas the HFHS diet contributed to the initiation of fibrosis. Our work provides the first line of evidence that diets rich in sucrose and fat induce oxidative damage in a peroxisomal FAO-dependent manner and not via mitochondria. Additionally, elevated fat intake, rather than elevated sucrose intake, contributes to the impairment of hepatic autophagic flux in the step of autolysosome acidification.

## 2. Materials and Methods

**Chemicals.** All reagents were obtained from Sigma-Aldrich (St. Louis, MO, USA).

**Ethics.** The studies presented in the paper were approved by Local Ethical Committees (Resolution No. 200/2016 on December, 11, 2016). All in vivo studies were performed according to the January, 15, 2015 Act on the Protection of Animals Used for Scientific Purposes in Poland, which follows the Directive 2010/63/EU of the European Parliament.

**Animals and dietary regimen.** Four-week-old male C57BL/6J mice (20–30 g) were purchased from CMD Bialystok (Bialystok, Poland). The mice were randomly divided into four groups, and each group was fed a standard chow (CHOW), HF, HS or HFHS ad libitum starting at the age of seven weeks. The standard chow diet derives 9% of its energy from fat, 58% from carbohydrates and 33% from protein and is composed of 3% fat and 5% sucrose, as described in Supplemental Table S1. The HF diet derives 51% of its energy from fat, 26% from carbohydrates and 23% from protein and is composed of 30% fat (14% SFAs, 12% monounsaturated fatty acids (MUFAs) and 1% polyunsaturated fatty acids (PUFAs)), 16% sucrose and 284 mg/kg cholesterol (Supplemental Table S1). The HS diet was composed of the CHOW diet supplemented with 30% sucrose in drinking water. The HFHS diet was composed of a HF diet supplemented with a HS diet. The CHOW (V1534) and HF (E15126) diets were purchased from Ssniff (Soest, Germany). The group of mice fed the CHOW diet was established as the control group of the experiment. All mice were housed in laboratory cages at 21–23 °C with 50–60% humidity (10–15 exchanges per h) with tap water and diet provided ad libitum for sixteen weeks. The mice were subjected to isoflurane inhalation anesthesia and then sacrificed by cervical dislocation. Systemic blood was immediately collected into EDTA KE-coated microtubes (Sarsted, Nümbrecht, Germany) and centrifuged at 2000 × *g* for 10 min. The plasma was isolated and stored for further analysis (−80 °C).

The livers were excised, weighed, and divided either for fixation in paraformaldehyde embedded in matrix medium or stored (−80 °C) until subsequent analysis.

**Aminotransferase (ALT) and aspartate aminotransferase (AST) levels.** ALT and AST levels were measured in the plasma with the use of a Reflotron® Plus System (Roche Diagnostics, Basel, Switzerland).

**Mitochondria isolation.** Livers (after excision of the gallbladder) were collected, minced, and homogenized in an ice-cold homogenization solution (50 mM Tris-HCl, pH 7.4, 75 mM sucrose, 225 mM mannitol, 0.5 mM EGTA, 0.5% essentially FA-free BSA). The homogenate was centrifuged for 3 min at 740× *g* and 4 °C. Then, the supernatant was collected and centrifuged for 5 min at 740× *g* and 4 °C and again for 10 min at 10,000× *g* and 4 °C. The resulting supernatant was collected as the cytosolic fraction. The mitochondrial pellet was resuspended in isolation solution II (50 mM Tris-HCl, pH 7.4, 75 mM sucrose, 225 mM mannitol, 0.5% essentially FA-free BSA) and centrifuged for 10 min at 10,000× *g* and 4 °C. The resulting mitochondrial pellet was resuspended in isolation solution III (50 mM Tris-HCl, pH 7.4, 75 mM sucrose, 225 mM mannitol) and centrifuged for 10 min at 10,000× *g* and 4 °C. The final mitochondrial pellet was resuspended in isolation solution III and kept on ice for the following measurements. Protein content was quantified according to the Bradford method.

**Measurement of oxygen consumption.** Mitochondrial oxygen consumption was measured in a Clark electrode (5300A Biological Oxygen Monitor; YSI, Rye Brook, NY, USA). The measurement was performed in a chamber with a medium composed of 50 mM Tris-HCl, pH 7.4, 225 mM mannitol, 75 mM sucrose, 0.5 mM EGTA, 1 mM MgCl_2_, 10 mM KCl, 5 mM glutamate and 5 mM malate at 25 °C. A total of 1 mg of fresh isolated mitochondria was added to the chamber. Basal respiration (State IV) was measured, followed by stimulated respiration (State III) measurement in the presence of 0.5 mM ADP. The respiratory control ratio (RCR) was calculated as the ratio of State III to State IV.

**Measurement of mitochondrial membrane potential.** Mitochondrial membrane potential was measured in a Shimadzu model RF 5000 spectrofluorometer (Shimadzu Corporation, Kyoto, Japan) at 495 nm and 586 nm excitation and emission wavelengths, respectively. The measurement was performed in a cuvette by adding 1 mg of fresh isolated mitochondria to the medium composed of 50 mM Tris-HCl (pH 7.4), 225 mM mannitol, 75 mM sucrose, 0.5 mM EGTA, 1 mM MgCl_2_, 5 mM glutamate, 5 mM malate and 8.3 µM safranin-O. The maximal mitochondrial membrane potential was considered the basal developed potential, and the minimal mitochondrial membrane potential was estimated in the presence of 2 µM FCCP.

**Measurement of ROS production.** ROS production was analyzed in the presence of 5 µM Amplex Red, 20 U/mL horseradish peroxidase and 40 U/mL superoxide dismutase (SOD) in a microplate reader (Infinite M200, Tecan, Männedorf, Switzerland) at an excitation wavelength of 560 nm and an emission wavelength of 590 nm. The measurement was initiated by adding 0.1 mg of freshly isolated mitochondria to the medium containing 50 mM Tris-HCl (pH 7.4), 75 mM sucrose, 225 mM mannitol and 5 mM glutamate/5 mM malate as the source of the substrate. A H_2_O_2_ standard curve was used to calibrate the method.

**Lipidomic analysis**

(a) Extraction of lipids. Lipids were extracted according to the Bligh and Dyer method. For the analysis, 1 mg of frozen isolated mitochondria and 2 mg of frozen liver tissue were used per sample. Samples were homogenized in a chloroform/methanol (2:1) mixture with 0.01% butylated hydroxytoluene (BHT). Two-phase systems (aqueous phase and organic phase) were obtained by adding distilled water, followed by vortexing the samples and centrifugation at 3000 rpm for 10 min at 4 °C. Lipid extracts were collected from the bottom phase of the centrifuged samples and stored until use at −20 °C.
(b) Mitochondrial phospholipid analysis. The organic phase of the lipid extracts was dried using a N_2_ flow. Lipid extracts redissolved in a chloroform/methanol (2:1) mixture were loaded on a thin-layer chromatography (TLC) silica gel-60 plate (Merck, Darmstadt, Germany), which was previously activated at 110 °C for 90 min. Phospholipids were developed in a TLC chamber with chloroform/methanol/acetic acid and water (50/37.5/3.5/2 (*v/v/v/v*)) as the mobile solvent for 2 h. To visualize phospholipid bands, the plate was soaked in 10% cupric sulfate/8% phosphoric acid and then burned at 140 °C for 20 min. The different classes of phospholipids were identified and quantified with ImageJ software (version 1.47).
(c) Liver neutral lipids analysis. The organic phase of the lipid extracts was dried using a N_2_ flow. Lipid extracts redissolved in a chloroform/methanol (2:1) mixture were loaded on a TLC silica gel-60 plate (Merck). Neutral lipids were developed in the heptane/isopropyl ether/glacial acetic acid (60/40/3 (*v/v/v*)) mobile phase for 50 min. The plate was soaked in 10% cupric sulfate/8% phosphoric acid and then burned at 140 °C for 20 min to visualize neutral lipid content. The different classes of neutral lipids were identified and compared to the phospholipid content (nonmobile phase-loading control). Quantification was performed by ImageJ software.
(d) Fatty acid analysis. Lipids were extracted according to the above-described sections. Briefly, total liver or mitochondrial lipids were separated on silica TLC plates with chloroform/methanol/acetic acid and water or heptane/isopropyl ether/glacial acetic acid for phospholipids and neutral lipids, respectively. Bands were visualized under an ultraviolet lamp after spraying with 0.2% 2,3-dichlorofluorescein and incubation with NH_3_. Bands were scraped off and incubated in a chloroform/methanol (4:1) mixture with 0.01% BHT overnight. FAs were transmethylated in the presence of 14% boron trifluoride in a methanol solution. Two-phase systems were obtained after adding hexane and water, followed by centrifugation at 3000 rpm for 10 min. The fatty acid methyl esters recovered from the upper phase were dried and resuspended in hexane. Analysis was performed by gas chromatography-mass spectrometry (GC-MS) with an Agilent 7890A-5975C GC-MS system (Agilent Technologies, Santa Clara, CA) equipped with an Agilent 19091N-205 capillary column. Nonadecanoic acid was used as an internal standard. The mass-to-charge ratios of the FA methyl esters were determined by selected ion monitoring.

**Lipid peroxidation analysis.** The levels of lipid peroxidation products were evaluated using a lipid peroxidation (malondialdehyde, MDA) assay kit (ab118970, Abcam, Cambridge, UK). Tissue (10 mg) and mitochondrial samples (500 µg) were homogenized according to the manufacturer’s protocol, with MDA-thiobarbituric acid (TBA) adducts formed by the addition of TBA solution. MDA standards were prepared in parallel with the samples. The TBA-MDA adducts were quantified colorimetrically by measuring the optical density at 532 nm.

**Oxidatively modified protein analysis.** The levels of carbonylated proteins were estimated using a protein carbonyl assay kit (ab178020, Abcam). Liver homogenates and mitochondrial samples (20 μg) were prepared according to the manufacturer’s protocol and separated on a 10% SDS polyacrylamide gel. By reaction with 2,4-dinitrophenylhydrazine (DNPH), dinitrophenyl (DNP)-derivatized proteins were visualized by incubation with the anti-DNP antibody followed by incubation with IRDye® 800CW secondary antibody (LI-COR Biosciences, Lincoln, NE, USA). Levels of protein carbonyl groups were normalized with the anti-VDAC1 antibody (ab34726, Abcam) in the mitochondrial fraction and with the anti-actin antibody (A5441, Sigma-Aldrich) in tissue homogenate.

The nitration of proteins was analyzed using a nitrotyrosine antibody (189542, Cayman Chemical, Ann Arbor, MI, USA). Briefly, 50 µg of mitochondrial fraction and total liver homogenate were loaded into a 10% SDS polyacrylamide gel. Nitrotyrosine proteins were detected by incubation with the antinitrotyrosine antibody followed by incubation with IRDye® 800CW secondary antibody (LI-COR Biosciences). Levels of nitrotyrosine were normalized with anti-VDAC1 in the mitochondrial fraction and with anti-actin in the tissue homogenate.

**Western blot analysis.** 50 mg of liver tissue samples were homogenized in 0.5 mL of cold lysis buffer (50 mM Tris, pH 8.0, 150 mM NaCl, 1% IGEPAL, 0.5% sodium deoxycholate, 0.1% SDS) supplemented with protease and phosphatase inhibitor cocktail immediately before use (1861281, Thermo Fisher Scientific, Waltham, MA, USA). Cytosolic and mitochondrial fractions were treated with a lysis buffer to guarantee proper protein extraction. Samples were incubated on ice for 15 min and centrifuged at 14,000× *g* for 20 min at 4 °C. Protein concentration was determined by the Bradford method. Protein samples (20–100 µg/lane) were prepared with reducing Laemmli loading buffer and denatured at 95 °C for 5 min according to the requirements of the antibody. Protein samples were separated on 10–12% polyacrylamide gels and transferred to polyvinylidene fluoride (PVDF) membranes. Membranes were blocked using Odyssey Blocking buffer (Li-Cor, Biosciences), followed by incubation with specific primary antibodies overnight. Then, the membranes were reincubated with appropriate secondary antibodies labeled with IRDye (Li-Cor, Biosciences). The relative levels of the detected protein on the membranes were visualized using an Odyssey infrared imaging system (Li-Cor Biosciences with fluorescent objectives). The fluorescence intensity of the membranes was analyzed using Image™ Studio software version for Odyssey® 3021, which is compatible with the Odyssey infrared imaging system. Primary antibodies against the following proteins were used: Akt1 (#2938, Cell Signaling, Danvers, MA, USA), LC3 (#12741, Cell Signaling), nitrotyrosine (sc81482, Santa Cruz Biotechnology, Dallas, TX, USA), OXPHOS cocktail (ab110413, Abcam) and phospho-Akt (Ser473) (#9271, Cell Signaling). The protein amount in each well was normalized to β-actin (A5441, Sigma-Aldrich) and VDAC (ab34726, Abcam) as a reference protein in the tissue/cytosolic and mitochondrial fractions, respectively.

**Evaluation of antioxidant enzyme activity.** Cytosolic and isolated mitochondrial fractions were obtained from frozen liver samples by differential centrifugation. Samples were prepared in ice-cold lysis buffer (50 mM Tris (pH 8.0), 150 mM NaCl, 1% IGEPAL, 0.5% sodium deoxycholate) and centrifuged at 16,000× *g* for 15 min at 4 °C. Antioxidant activities were evaluated according to the manufacturer’s instructions for each assay kit. SOD activity was evaluated according to the SOD assay kit (ab65354, Abcam) by measuring the optical density at 450 nm. Catalase activity was assessed using a catalase assay kit (707002, Cayman Chemical) by measuring the absorbance at 540 nm. Glutathione peroxidase (GPX) activity was assessed by a GPX assay kit (ab102530, Abcam), while glutathione reductase (GR) was evaluated by a GR assay kit (703202, Cayman Chemical). The activities of both GPX and GR were measured at an optical density of 340 nm.

**Blue Native (BN) PAGE.**

(a) Sample preparation. Frozen liver samples were homogenized, and mitochondria were isolated according to the protocol described above in the mitochondria isolation methodology section. Thereafter, mitoplast pellets were resuspended in ACBT buffer (75 mM Bis-Tris, pH 7.0, 1.5 mM ɛ-aminocaproic acid) and 20% β-lauryl maltoside and incubated on ice for 10 min. The samples were centrifuged at 10,000× *g* for 30 min at 4 °C, and then, the supernatant was collected. Protein concentration was measured by the Bradford method. Thereafter, BN sample buffer (50 mM Bis-Tris, pH 7.0, 750 mM aminocaproic acid, 0.5 mM EDTA and 5% Serva Blue G) (1:10) was added to the native mitochondrial lysate.
(b) Nondenaturing PAGE. Next, freshly prepared native mitochondrial lysate samples (20 µg/lane) were loaded and separated on a 4–15% polyacrylamide native gel. To visualize optimal separation of OXPHOS complexes, mouse heart mitochondria (10 µg) sample was used as an internal standard. The gel ran with a constant voltage of 75 V until the blue front migrated to the middle of the gel. Then, cathode buffer B (blue) was replaced by cathode buffer A. Then, the voltage was increased to 150 V until the blue front reached the end of the gel.

In-gel activity assay. To evaluate Complex I activity, the BN-PAGE gel was incubated in a solution containing 3 mM Tris-HCl (pH 7.4), 113 µM β-nicotinamide adenine dinucleotide (NADH), and 245 µM of nitro tetrazolium blue chloride (NTB) for 20 min at 37 °C. To assess Complex II activity, the BN-PAGE gel was incubated in a solution containing 1.5 mM phosphate buffer, pH 7.4, 5 mM EDTA, 0.8 mM KCN, 50 mM succinate, 0.2 mM phenazine methosulphate and 245 µM NTB for 20 min at 37 °C. To evaluate Complex IV activity, the BN-PAGE gel was incubated in a solution containing 30 mM phosphate buffer (pH 7.4), 0.24 M sucrose, 2.3 mM diamino benzidine tetrachloride (DAB), 24 mM cytochrome c and 0.04 mg/mL catalase for 1–2 h at 37 °C. To visualize Complex V activity, the BN-PAGE gel was incubated overnight at 37 °C in a solution containing 35 mM Tris-HCl, pH 7.8, 0.3 M glycine, 14 mM MgSO_4_.7H20, 9 mM ATP and 3.7 mM Pb_2_(NO_3_)_2_.

**Statistical analysis.** Data are expressed as the mean ± standard error of the mean (SEM). All statistical tests were performed using GraphPad Prism version 8.0.2 for Windows (GraphPad Software, Inc., San Diego, CA USA). Normality was determined by the Shapiro–Wilk normality test. Non-normally distributed datasets were analyzed using the nonparametric Mann–Whitney test (two groups) or Kruskal–Wallis test (more than two groups) followed by Dunn’s test for multiple comparisons. Parametric tests using Student’s *t*-test and one-way ANOVA were performed on normally distributed datasets followed by Welch’s correction or Bonferroni’s multiple comparisons test, respectively. The level of significance was *P* < 0.05.

A detailed description of liver histology and mass spectrometry analyses can be found in the Supplementary Materials.

## 3. Results

**Figure 1:**
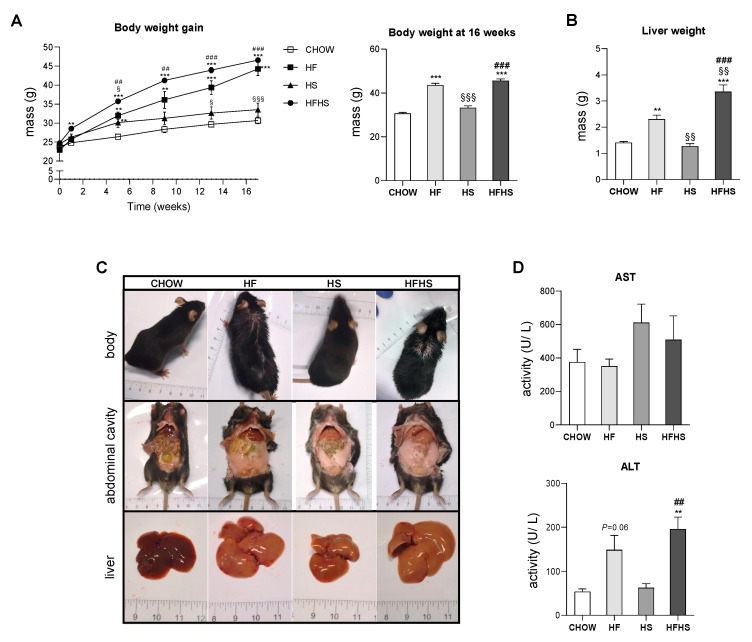
HFHS diet causes body and liver weight gain and increases ALT levels. (**A**) Body weight gain profile during the 16-week feeding period (left panel) and body weight at the end of the feeding period (right panel) (*N* = 18). (**B**) Liver weight at the end of the feeding period (*N* = 4). (**C**) Representative images of body, abdominal cavity and liver appearance at the end of the feeding period. (**D**) Plasma AST and ALT levels (*N* = 4). All data are expressed as the mean ± SEM. (§) vs. the HF diet (*P* < 0.05); (**) vs. the CHOW diet, (§§) vs. the HF diet and (##) vs. the HS diet (*P* < 0.01); (***) vs. the CHOW diet, (§§§) vs. the HF diet and (###) vs. the HS diet (*P* < 0.001); *P* values were determined using one-way ANOVA followed by Bonferroni’s post hoc test. HF, high-fat; HS, high-sucrose; HFHS, high-fat plus high-sucrose; AST, aspartate aminotransferase; ALT, alanine aminotransferase.

**HFHS intake is associated with body and liver weight gain and hepatocyte damage.** Intake of the HF or HFHS diet, but not the HS diet, progressively increased the body weight of the mice during the 16-week feeding period (Figure 1A), accompanied by an increase in liver weight, most prominently in the HFHS-diet-fed mice (Figure 1A,B). All high-caloric diets caused visceral fat accumulation (Figure 1C). Moreover, we observed more discolored livers in all treated groups, suggesting steatosis. The increased ALT levels in the HF- and HFHS-diet-fed animals indicated hepatocyte death (Figure 1D).

**Figure 2:**
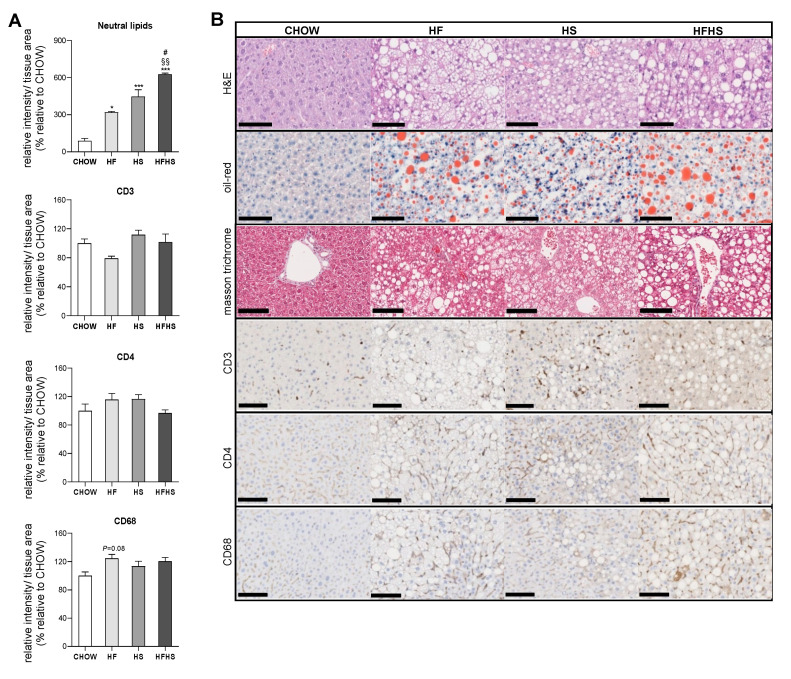
HF, HS and HFHS diets induce simple steatosis with no signs of lobular inflammation. (**A**) Hepatic content of neutral lipids and CD3, CD4 and CD68 inflammatory markers obtained from three independent images (per animal) from the experimental conditions shown in (**B**) (*N* = 4). (B) Representative images of paraffin-embedded liver sections with H&E, Oil Red O, Masson’s trichrome, CD3, CD4 and CD68 immunostaining. Scale bar, 250 µm; magnification, 10×. All data are expressed as the mean ± SEM. (*) vs. the CHOW diet and (#) vs. the HS diet (*P* < 0.05); (§§) vs. the HF diet (*P* < 0.01); (***) vs. the CHOW diet (*P* < 0.001); *P* values were determined using one-way ANOVA followed by Bonferroni’s post hoc test. H&E, hematoxylin and eosin; HF, high-fat; HFHS, high-fat plus high-sucrose; HS, high-sucrose.

**Steatosis is caused by prolonged consumption of a HF or HS diet but was aggravated to fibrosis initiation by the combined HFHS diet.** Established by Kleiner et al., the NAFLD activity score (NAS) grades NAFLD according to three main criteria: steatosis, inflammation and fibrosis [18]. As previously reported [19], an increase in body mass index is well correlated with the development of steatosis, as this stage is diagnosed by the presence of fat in at least 5% of hepatocytes. According to the NAS criteria, the HF, HS and HFHS diets cause similar levels of steatosis (Grade 3: > 66%) (Supplemental Table S3). A detailed analysis of Oil Red O staining revealed that among the diets, the HFHS diet was associated with the most pronounced fat accumulation (six-fold increase) in the liver. Additionally, the HF and HFHS diets were associated with macrosteatosis, whereas microsteatosis was more apparent in the HS-diet-fed mice (Figure 2A,B). Decreased levels of glycogen were found in all experimental groups with hypercaloric diets compared with the CHOW group (Supplemental Figure S1A,B).

All high-caloric diets were associated with a similar degree of hepatocyte ballooning (Grade 2, Supplemental Table S3). Although, no significant signs of CD3 and CD4 inflammatory markers and just a few signs of CD68-positive cell aggregation observed in the HF-diet-fed mice (Figure 2A) confirmed the absence of lobular inflammation (Grade 0, Supplemental Table S3) in the hypercaloric-diet-fed mice. Masson’s trichrome staining demonstrated signs of perisinusoidal fibrosis in the HFHS-diet-fed mice (Figure 2B and Supplemental Table S3). Additionally, a decrease in the deposition of type 3 collagen reticular fibers in the HF- and HFHS-diet-fed mice suggested alterations in the normal architecture of hepatic cells (Supplemental Figure S1C,D).

Thus, the HF, HS and HFHS diets induced NAFL characterized by steatosis, without significant inflammation, but signs of early fibrosis (NASH) were observed in the HFHS diet group after 16 weeks of feeding.

**Figure 3:**
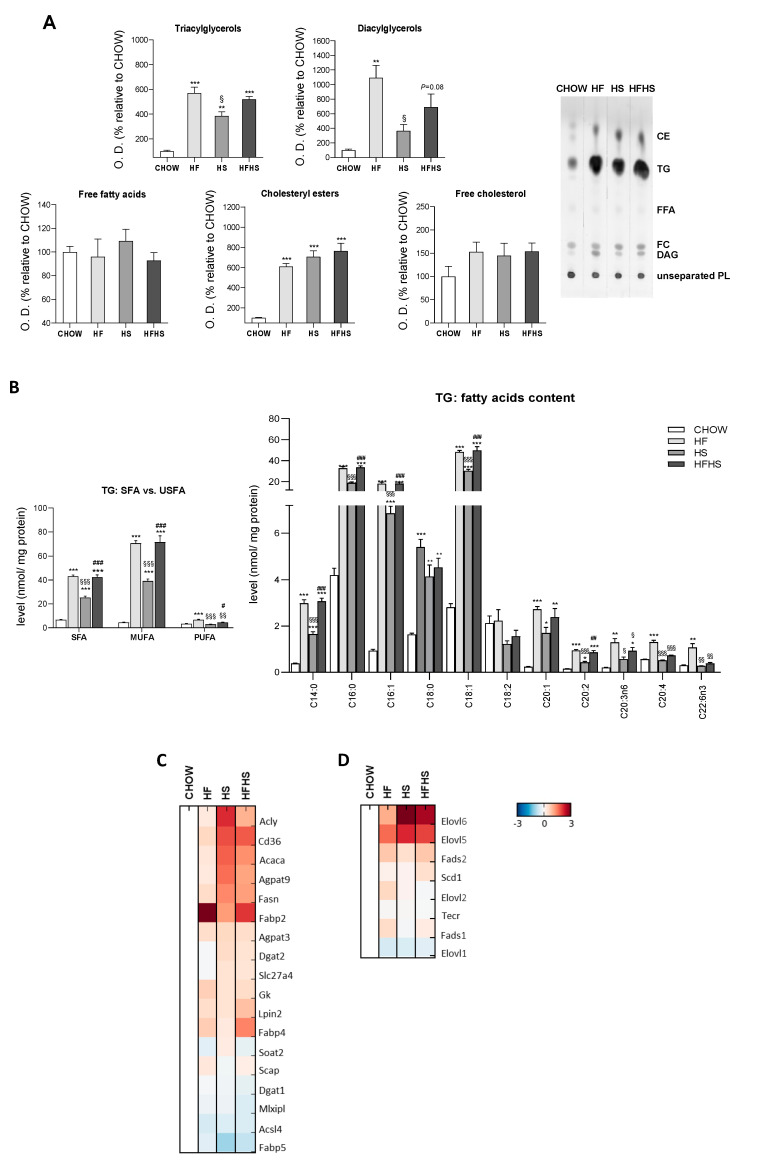
HF, HS and HFHS diets increase hepatic lipid accumulation in the form of TGs enriched in SFAs and MUFAs. (**A**) Optical density (arbitrary units) of hepatic tissue content of TGs, diacylglycerols, free FAs, CEs and free cholesterol and representative thin-layer chromatography image of a replicate showing all neutral lipid species (*N* = 4). (**B**) Levels of SFAs, MUFAs and PUFAs in the hepatic tissue TGs fraction and detailed analysis of FAs acyl chains in TGs (*N* = 4). (**C**) Proteomic analysis of protein levels involved in the de novo lipogenesis (DNL) pathway (*N* = 4). (**D**) Proteomic analysis of protein levels involved in the elongation and desaturation of FAs (*N* = 4). Blue represents decreased levels, and red represents increased levels (*N* = 4). All data are expressed as the mean ± SEM. (*) vs. the CHOW diet, (§) vs. the HF diet and (#) vs. the HS diet (*P* < 0.05); (**) vs. the CHOW diet, (§§) vs. the HF diet and (##) vs. the HS diet (*P* < 0.01); (***) vs. the CHOW diet, (§§§) vs. the HF diet and (###) vs. the HS diet (*P* < 0.001); *P* values were determined using one-way ANOVA followed by Bonferroni’s post hoc test. CE, cholesteryl ester; DG, diacylglycerol; FC, free cholesterol; FFA, free fatty acids; HF, high-fat; HFHS, high-fat plus high-sucrose; HS, high-sucrose; PL, phospholipid; PUFA, polyunsaturated fatty acid; SFA, saturated fatty acid; TG, triacylglycerol; USFA, unsaturated fatty acid.

**Fat and sucrose induce the synthesis of triacylglycerols** (**TGs) significantly enriched with MUFAs.** The majority of the lipid accumulation in the livers of mice fed the HF, HS and HFHS diets was in the form of TGs and cholesteryl esters (CEs) (Figure 3A). Moreover, all hypercaloric diets also induce increased levels of diacylglycerols (Figure 3A). Furthermore, we observed increased levels of proteins involved in the DNL pathway, namely, ATP-citrate synthase (ACLY), acetyl-CoA carboxylase 1 (ACACA), fatty acid synthase (FASN) and glycerol-3-phosphate acyltransferase 3 (AGPAT9), in the HS- and HFHS-treated groups (Figure 3C). Accordingly, increased carbohydrate availability (HS diet) contrasts with the composition of the HF diet in which DNL was found unchanged.

Despite the similar content of SFAs (14%) and unsaturated fatty acids (USFAs) (12%) in the HF diet (Supplemental Table S1), MUFAs were highly increased in the accumulated hepatic TGs in the HF- and HFHS-diet-fed mice (Figure 3B). Although to a lower extent, similar tendencies were observed in the HS-diet-fed group. Increased accumulation of SFAs (C14:0, C16:0 and C18:0) was found in the hepatic TGs of all treated mouse groups compared with the CHOW group (Figure 3B). Moreover, the C16:1, C18:1 and C20:1 MUFA species were especially highly abundant relative to PUFAs, such as C20:2, C20:3n6, C20:4 and C22:6n3, which were only slightly elevated in the HF- and/or HFHS-diet-fed mice compared with the CHOW-fed mice (Figure 3B). MUFAs abundance was associated with an elevated content of elongases (elongation of very-long-chain fatty acids protein (ELOVL) 5/6) and desaturases (acyl-CoA desaturase 1 (SCD-1) and fatty acid desaturase (FADS) 2), while the low content of ELOVL1/2 and FADS1 correlated with no significant enrichment in PUFAs (Figure 3D). These changes indicate substantial similarities in FAs composition in the liver lipid droplets of the HF- and HS-diet-fed mice.

**Figure 4:**
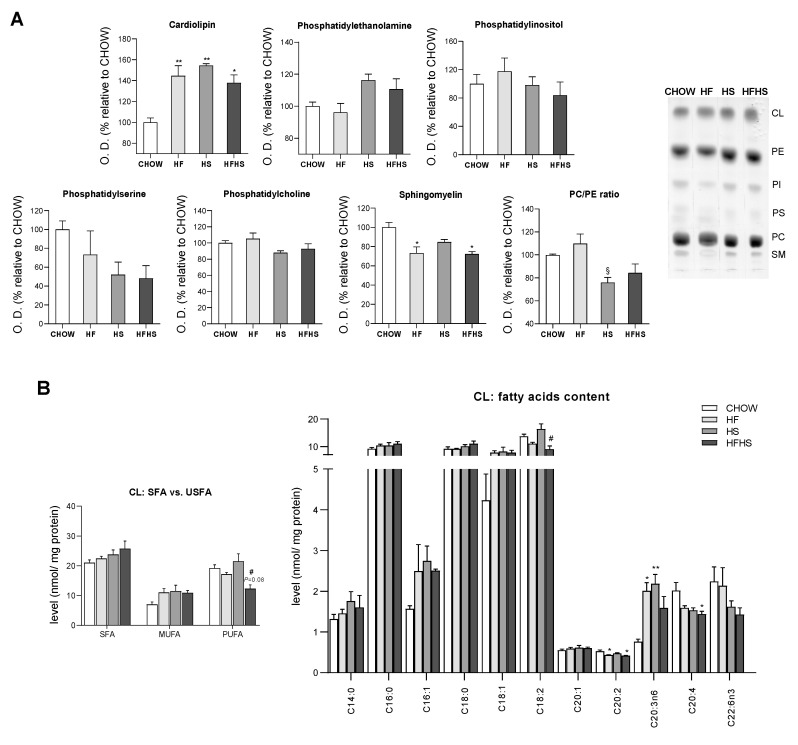
HF, HS and HFHS diets trigger mitochondrial phospholipid remodeling. (**A**) Optical density (arbitrary units) of mitochondrial content of CL, PE, PI, PS, PC and SM and the calculated PC/PE ratio. Representative thin-layer chromatography image of a replicate showing all phospholipid species (*N* = 4). (**B**) CL content of SFAs, MUFAs and PUFAs in isolated hepatic mitochondria and detailed analysis of FA acyl chains in CL (*N* = 4). All data are expressed as the mean ± SEM. (*) vs. the CHOW diet, (§) vs. the HF diet and (#) vs. the HS diet (*P* < 0.05); (**) vs. the CHOW diet (*P* < 0.01); *P* values were determined using one-way ANOVA followed by Bonferroni’s post hoc test or Kruskal–Wallis test followed by Dunn’s post hoc test. The purity of the mitochondrial fraction is shown in Figure 7D. CL, cardiolipin; HF, high-fat; HFHS, high-fat plus high-sucrose; HS, high-sucrose; PC, phosphatidylcholine; PE, phosphatidylethanolamine; PI, phosphatidylinositol; PS, phosphatidylserine; PUFA, polyunsaturated fatty acid; SFA, saturated fatty acid; SM, sphingomyelin; USFA, unsaturated fatty acid.

**Liver mitochondria from HS- and HFHS-diet-fed mice are enriched in cardiolipin (CL) but show reduced phosphatidylcholine (PC)/phosphatidylethanolamine (PE) ratio.** Due to the central role of mitochondria in lipid metabolism, we next analyzed the lipidomic profile of liver mitochondria from mice fed the experimental diets. While no significant alterations were observed in their PC content, increased levels of PE were found in the liver mitochondrial membranes of the HS- and HFHS-diet-fed mice compared with those of the CHOW group (Figure 4A). The levels of both major constituents of mitochondrial membranes are used to calculate the PC/PE ratio, an important indicator of mitochondrial membrane fluidity and integrity. A decreased PC/PE ratio in the liver mitochondria of the HS- and HFHS-diet-fed mice compared with those of the CHOW-diet-fed mice was observed (Figure 4A), indicating the maintenance of membrane fluidity. The relative abundance of SFAs/USFAs in PC and PE was measured. PUFAs were decreased in PC (Supplemental Figure S2B) but tended to be increased in PE in the HS and HFHS groups compared with the CHOW group (Supplemental Figure S2A). According to the HF diet composition, the HF and HFHS diets reduced the abundance of 18:2 in both phospholipids (Supplemental Table S1 and Figure S2A,B).

Compared with the CHOW diet, all the hypercaloric diets increased liver mitochondria cardiolipin (CL) levels by 40 to 50% (Figure 4A) and altered the saturation/unsaturation levels of the CL acyl chains. While no major changes in SFAs were observed, MUFAs (C16:1 and C18:1) were enriched in all treated groups (Figure 4B). Interestingly, reduced levels of PUFAs, including C18:2, the most abundant, C20:2, C20:4 and C22:6n3, were found in the HFHS group compared with the CHOW group (Figure 4B). Regarding phospholipids present at low levels in mitochondrial membranes, phosphatidylinositol (PI) remained unaltered, while a downward trend in phosphatidylserine (PS) was observed in the HS and HFHS groups (Figure 4A). Moreover, a significant decrease in sphingomyelin (SM) was detected in the liver mitochondria of the HF- and HFHS-diet-fed mice compared with those of the CHOW-diet-fed mice (Figure 4A). Thus, all hypercaloric diets induced changes in the mitochondrial phospholipid profile, including in FAs, after a 16-week feeding period.

**Figure 5:**
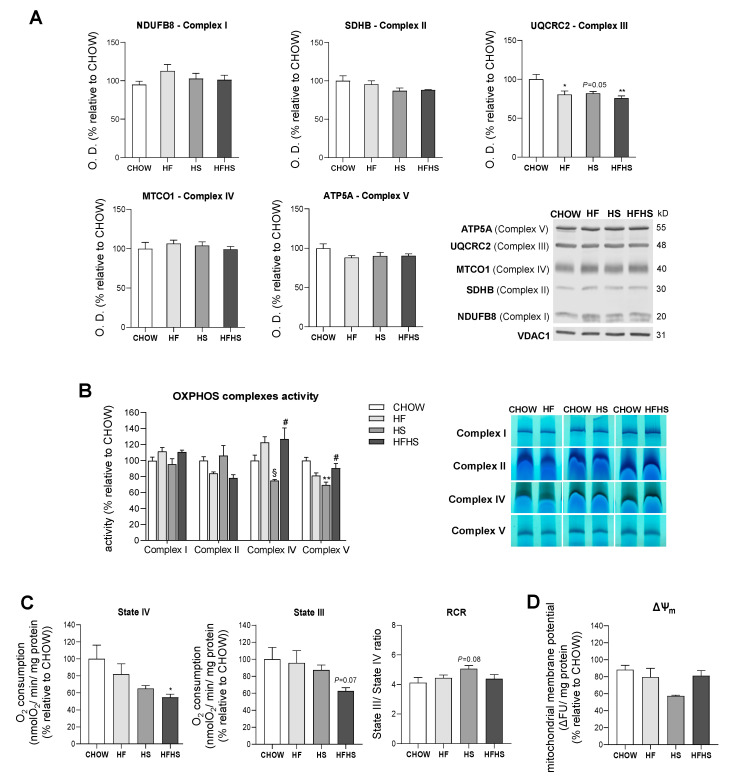
The HFHS diet decreases mitochondrial respiration. (**A**) Optical density (arbitrary units) of OXPHOS subunits (NDUFB8, Complex I; SDHB, Complex II; UQCRC2, Complex III; MTCO1, Complex IV; and ATP5A, Complex V) levels and representative Western blot images. VDAC1 was used as a loading control (*N* = 4). (**B**) OXPHOS complex activities and in-gel activities representative image (*N* = 4). (**C**) Oxygen consumption rate in the basal state (State IV) and in the ADP-stimulated state (State III) and RCR (State III/State IV) (*N* = 6). (**D**) Mitochondrial membrane potential (*N* = 8). All data are expressed as the mean ± SEM. (*) vs. the CHOW diet, (§) vs. the HF diet and (#) vs. the HS diet (*P* < 0.05); (**) vs. the CHOW diet (*P* < 0.01); *P* values were determined using one-way ANOVA followed by Bonferroni’s post hoc test or Kruskal–Wallis test followed by Dunn’s post hoc test. ΔΨ_m_, mitochondrial membrane potential; HF, high-fat; HFHS, high-fat plus high-sucrose; HS, high-sucrose; RCR, respiratory control ratio.

**HF and HS diets show small effects on mitochondrial OXPHOS complexes abundance and activities, while the HFHS diet changes mitochondrial respiration.** Altered phospholipidic compositions of mitochondrial membranes may compromise membrane integrity and permeability and hence could affect the function of mitochondrial electron transport chain (ETC) complexes in the inner mitochondrial membrane (IMM) [20]. In fact, we observed a significant decrease of 18 to 25% in UQCRC2 (Complex III subunit) levels, while a small, although not statistically significant, decrease was found for SDHB (Complex II subunit) and ATP5A (Complex V subunit) in all the modified diets compared with the CHOW diet (Figure 5A). A comparative mass spectrometry (MS)-based proteomic analysis revealed a global downward trend in the levels of subunits/assembly factors of all OXPHOS complexes (Complexes I–V) in the HS- and HFHS-diet-fed mice compared with the CHOW-fed mice (Supplemental Figure S3A–E). In-gel activity assays indicated decreased activities of Complex II in the HF and HFHS diets, Complex IV in the HS diet and Complex V in the HF and HS diets compared with the CHOW diet (Figure 5B). It is important to highlight that activities of OXPHOS complexes were assessed independently when individual complexes were taken from supercomplexes structures and from the lipid environment. These alterations in OXPHOS subunit levels and in the activities of assembled complexes suggested an impairment of mitochondrial function in the livers of the HF-, HS- and HFHS-diet-fed mice. Accordingly, the measured mitochondrial oxygen consumption in both the basal (State IV) and ADP-stimulated states (State III) was decreased 46% and 38%, respectively, in the HFHS-diet-fed mice compared with the CHOW group (Figure 5C). A slight increase in the RCR in the HS group compared with the CHOW-fed mice was indicative of a higher capacity to convert ADP in ATP under this condition (Figure 5C). Furthermore, we also assessed the capacity of mitochondria to generate their membrane potential (ΔΨm). Although very similar values were found between the CHOW and the HF or HFHS groups, ΔΨm was diminished in the HS-diet-fed mice (Figure 5D). Interestingly, the alterations in OXPHOS complex levels in the HF and HS groups did not induce significant changes in mitochondrial OXPHOS efficiency; however, the HFHS diet was associated with a decrease in mitochondrial respiration.

**Figure 6:**
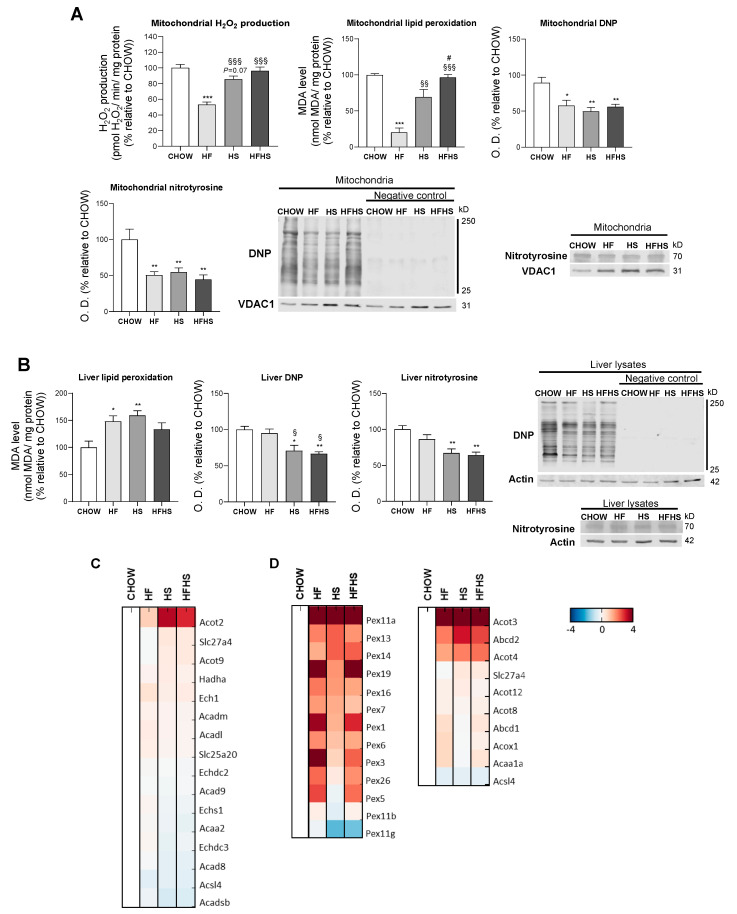
Hepatic oxidative damage with no signs of mitochondrial ROS production in diet-induced nonalcoholic fatty liver (NAFL). (**A**) Rate of mitochondrial H_2_O_2_ production, levels of mitochondrial lipid peroxidation and optical density (arbitrary units) of mitochondrial protein oxidative damage (DNP and nitrotyrosine), with representative Western blot images. VDAC1 was used as a loading control (*N* = 4). (**B**) Levels of lipid peroxidation and optical density (arbitrary units) of protein oxidative damage (DNP and nitrotyrosine) in hepatic tissue lysate, with representative Western blot images. Actin was used as a loading control (*N* = 4). (**C**) Proteomic analysis of protein levels involved in mt-FAO (*N* = 4). (**D**) Proteomic analysis of protein levels involved in peroxisomal FAO and in peroxisomal abundance (*N* = 4). Blue represents decreased levels, and red represents increased levels. Data are expressed as the mean ± SEM. (*) vs. the CHOW diet, (§) vs. the HF diet and (#) vs. the HS diet (*P* < 0.05); (**) vs. the CHOW diet and (§§) vs. the HF diet (*P* < 0.01); (***) vs. the CHOW diet and (§§§) vs. the HF diet (*P* < 0.001); *P* values were determined using one-way ANOVA followed by Bonferroni’s post hoc test. DNP, dinitrophenyl; HF, high-fat; HFHS, high-fat plus high-sucrose; HS, high-sucrose.

**Figure 7:**
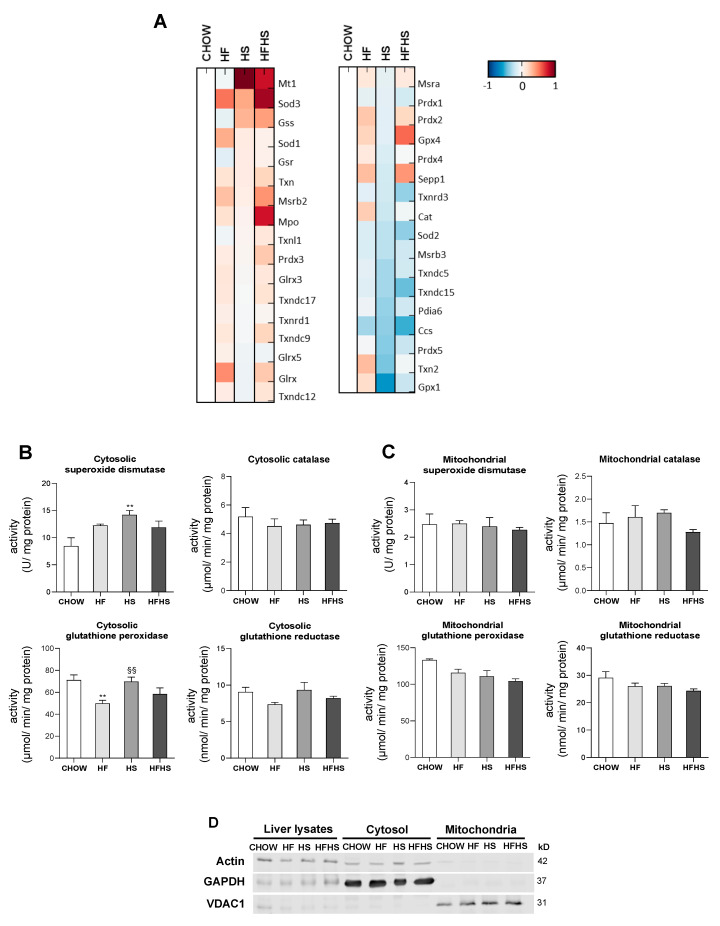
HF, HS and HFHS diets lead to an imbalance in antioxidant enzyme activities in the cytosolic fraction but not in the mitochondrial fraction. (**A**) Proteomic analysis of antioxidant enzyme levels in hepatic tissue (*N* = 4). Blue represents decreased levels, and red represents increased levels. (**B**) Antioxidant enzyme activities of superoxide dismutase (SOD), catalase, glutathione peroxidase (GPX) and glutathione reductase (GR) in the hepatic cytosolic fraction (*N* = 4). (**C**) Antioxidant enzyme activities of SOD, catalase, GPX and GR in the hepatic mitochondrial fraction (*N* = 4). (**D**) Representative Western blot images showing the purity of the hepatic cytosolic and mitochondrial fractions. GAPDH and VDAC1 were used as the cytosolic and mitochondrial loading controls, respectively. All data are expressed as the mean ± SEM. (**) vs. the CHOW diet and (§§) vs. the HF diet (*P* < 0.01); *P* values were determined using one-way ANOVA followed by Bonferroni’s post hoc test. GAPDH, glyceraldehyde-3-phosphate dehydrogenase; HF, high-fat; HFHS, high-fat plus high-sucrose; HS, high-sucrose; VDAC1, voltage-dependent anion channel 1.

**HF and HS diets reduce mitochondrial oxidative damage but induce hepatic oxidative damage in a peroxisomal FAO-dependent manner.** Next, we investigated whether the observed OXPHOS remodeling altered mitochondrial ROS production and manifested oxidative damage in either mitochondria or whole liver tissue. Surprisingly, HF induced a decrease in mitochondrial H_2_O_2_ production, while HS resulted in a nonstatistically significant decrease in the same parameter. Results from the HFHS-diet-fed mice were similar to the control. Accordingly, the mitochondrial lipid peroxidation caused by the modified diets exhibited the same profile as the changes in mitochondrial ROS production. The oxidative damage of mitochondrial proteins as evaluated by DNP and nitrotyrosine analysis was lower in the HF-, HS- and HFHS-diet-fed animals than in the CHOW-fed animals (Figure 6A). In contrast, lipid peroxidation was found to be increased by 50% in whole liver tissue from mice fed the HF and HS diets compared with that from mice fed the CHOW diet, while only a nonstatistically significant increase was observed in mice fed the HFHS diet. DNP and nitrotyrosine levels were also significantly lower in the HS- and HFHS-diet-fed mice than in the CHOW-fed mice, whereas the HF diet did not cause oxidative changes in the liver proteins (Figure 6B). As FAO is also involved in ROS formation, we also evaluated mt-FAO and peroxisomal FAO by MS-based proteomic analysis. Increased levels of hormone-sensitive lipase (LIPE) and monoglyceride lipase (MGLL) (data not shown), which are involved in TGs hydrolysis, indicated the upregulation of both mt-FAO and peroxisomal FAO by hypercaloric diets. Although acyl-coenzyme A thioesterase (ACOT)2 was enhanced in the HF-, HS- and HFHS-diet-fed mice, the levels of the other mt-FAO proteins in these groups were similar to those in the CHOW group (Figure 6C). In contrast, however, peroxisomal FAO-related proteins (ACOT3, ACOT4 and ATP-binding cassette subfamily D member 2 (ABCD2)) were strongly upregulated several-fold under the HF, HS and HFHS conditions (Figure 6D). In fact, an increase in peroxisomal FAO is correlated with a two- to four-fold increase in peroxisomal abundance-related proteins, as shown for peroxisomal biogenesis factors (PEX1 and PEX3), peroxisomal membrane proteins (PEX11a, PEX13, PEX14, PEX16) and peroxisomal assembly proteins (PEX6, PEX19) (Figure 6D). Since the antioxidant defense system is involved in ROS neutralization, we first assessed hepatic levels of antioxidant enzymes by MS-based proteomic analysis. Increased levels of metallothionein-1 (MT1) were induced by the HS and HFHS diets, with SOD-3 and selenoprotein P (SEPP1) enhanced by the HF and HFHS diets (Figure 7A). Compared with the CHOW diet, the HF diet resulted in increased glutaredoxin (GLXR) levels, while the HFHS diet increased myeloperoxidase (MPO) and GPX-4 levels (Figure 7A). Interestingly, we found an increase in cytosolic SOD activity according to the lipid peroxidation damage profile observed in livers. However, the lack of change in catalase activity (Figure 7B) indicates an imbalanced antioxidant defense system in the livers of animals fed the hypercaloric diets. The cytosolic activity of GPX was decreased in the HF-diet-fed mice compared with the CHOW-diet-fed mice, while a nonstatistically significant decrease in cytosolic GR was observed in the HF- and HFHS-diet-fed mice (Figure 7B). In contrast, in mitochondrial fractions, only a nonstatistically significant decrease, presenting a downward trend, in GPX and GR activities was shown in the three modified diets compared with the control diet (Figure 7C). No changes in the activity of mitochondrial SOD were observed. These data indicate that the hepatic oxidative damage induced by all of the hypercaloric diets may not be due to ROS originating from mitochondria but due to an imbalance between increased peroxisomal FAO that produces ROS and the cytosolic/cellular antioxidant capacity.

**Figure 8:**
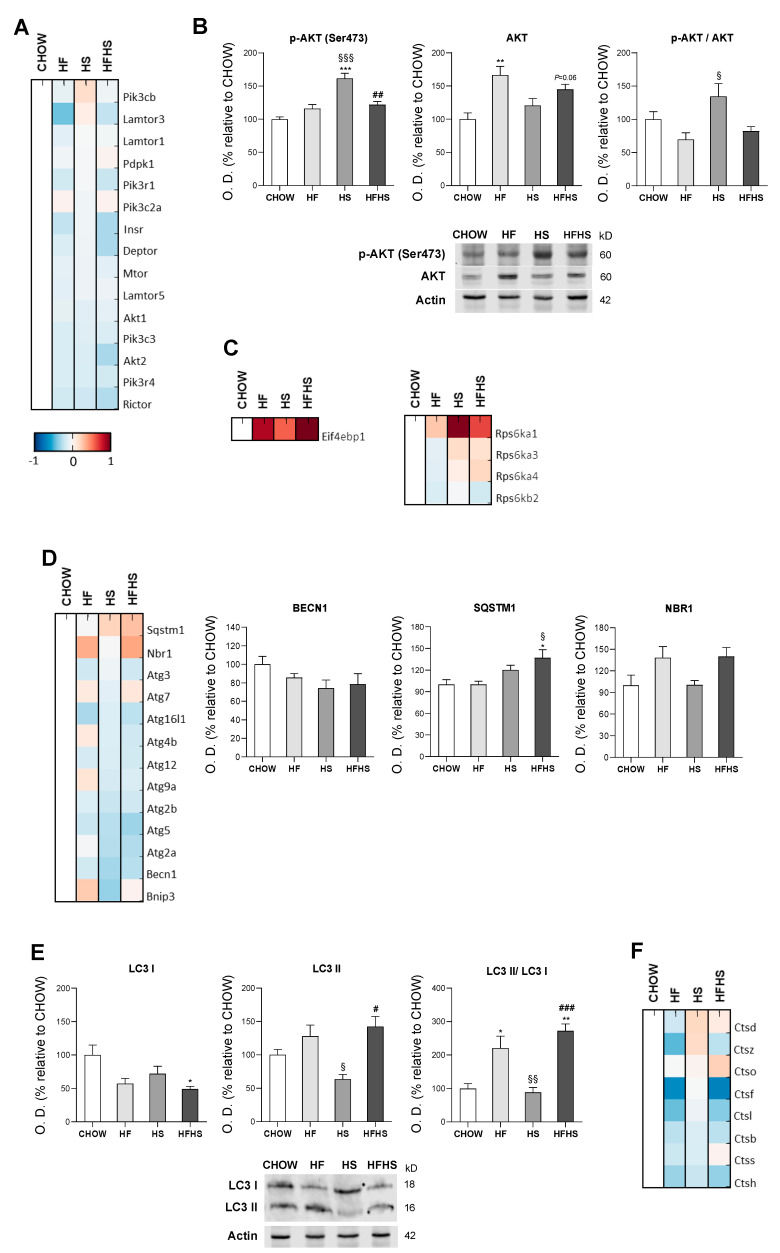
HF and HFHS diets induce SQSTM1 and LC3-II accumulation and reduce cathepsin levels. (**A**) Proteomic analysis of proteins in the PI3K/AKT/mTOR pathway (*N* = 4). (**B**) Optical density (arbitrary units) of Ser473 p-AKT, AKT and the p-AKT/AKT ratio, with representative Western blot images. Actin was used as a loading control (*N* = 4). (**C**) Proteomic analysis of proteins involved in protein translation and protein synthesis (*N* = 4). (**D**) Proteomic analysis of proteins involved in autophagosome formation and cargo capture in the autophagy pathway (*N* = 4). (**E**) Optical density (arbitrary units) of LC3-I, LC3-II and the LC3-II/LC3-I ratio, with representative Western blot images. Actin was used as a loading control (*N* = 4). (**F**) Proteomic analysis of cathepsin levels. Blue represents decreased levels, and red represents increased levels (*N* = 4). All data are expressed as the mean ± SEM. (*) vs. the CHOW diet, (§) vs. the HF diet and (#) vs. the HS diet (*P* < 0.05); (**) vs. the CHOW diet, (§§) vs. the HF diet and (##) vs. the HS diet (*P* < 0.01); (***) vs. the CHOW diet, (§§§) vs. the HF diet and (###) vs. the HS diet (*P* < 0.001); *P* values were determined using one-way ANOVA followed by Bonferroni’s post hoc test or Kruskal–Wallis test followed by Dunn’s post hoc test. BECN1, beclin-1; HF, high-fat; HFHS, high-fat plus high-sucrose; HS, high-sucrose; NBR1, next to BRCA1 gene 1; SQSTM1, sequestosome 1.

**HF, HS and HFHS diets affect the protein homeostasis network.** We evaluated PI3K/AKT/mTOR signaling as a key regulator of the cellular adaptive response to oxidative stress. Although MS-based proteomic analysis of the PI3K/AKT/mTOR axis showed no major changes (Figure 8A), phosphorylation of AKT at Ser473 and the p-AKT/AKT ratio indicated activation of this pathway in the HS-diet-fed mice. Compared with the CHOW-fed mice, the HF- and HFHS-diet-fed mice exhibited an upward trend in p-AKT (Ser473) together with increased total AKT levels (Figure 8B). Consistent with the activation of this pathway, increased levels of proteins involved in protein translation inhibition (EIF4EBP1) and protein synthesis (mainly RPS6KA1 and RPS6KA3) were found in the high-caloric diets (Figure 8C). Importantly, autophagy, a key cellular stress response and quality control mechanism (regulated by PI3K/AKT/mTOR signaling), appeared to be affected. MS-based proteomic and Western blot analyses showed the reduced expression of the main autophagic proteins (autophagy-related proteins (ATGs) and beclin-1 (BECN1)) in the HF-, HS- and HFHS-fed groups compared with the CHOW-diet-fed group. However, an increase of 120% and 170% in the autophagosome-associated LC3-II/LC3-I ratio and increased sequestosome-1 (SQST1M) and especially autophagy cargo receptor (NBR1) levels (which is involved in the recognition of ubiquitin-modified unknown peroxisomal membrane proteins) in the HF- and HFHS-diet-fed mice indicate the possible blockade of autophagosomal clearance (Figure 8D,E). In fact, this assumption is supported by a reduction in enzymes responsible for lysosomal acidification and, consequently, autolysosomal proteolysis. We observed decreased levels of cathepsin D in the livers of the HF-diet-fed mice and decreased levels of cathepsins B and L in the HF- and HFHS-diet-fed mice compared with the CHOW-diet-fed mice (Figure 8F). Thus, exposure to all hypercaloric diets affects cellular proteostasis, and fat seems to be harmful for the clearance of damaged organelles and biomolecules.

## 4. Discussion

Liver fat accumulation has been mostly attributed to the consumption of fat- and sucrose-enriched diets. Mechanisms such as oxidative stress, mitochondrial dysfunction, endoplasmic reticulum (ER) stress, inflammasome stimulation or apoptotic pathway activation have been suggested to be involved in the pathognomonic progression from "simple" steatosis to NASH [2]. However, many discrepancies, namely those related to mitochondrial structure and function, remain as to the exact sequence of events leading to the early developmental stage of NAFLD. Our results demonstrate that mitochondria-independent pathways contribute to hepatic damage in NAFL, mainly involving both peroxisomal FAO-induced oxidative damage and autophagy impairment. Autophagic flux blockade could play a role in accelerating fatty liver and lipotoxic injuries during NAFL.

Overweight with liver enlargement accompanied by increased ALT levels in response to HF and HFHS diets reflected the development of hepatic steatosis. Despite contributing to increased steatosis, lipid accumulation mostly in the form of TGs and CEs has been described to protect hepatocytes from the lipotoxic effects of free FAs and FAs-lipid intermediates. Accordingly, Yamaguchi et al. showed that steatosis protects the liver from progressive damage associated with oxidative stress, inflammation and fibrosis, markers that have been linked to NASH [21]. Intrahepatic lipid accumulation is mainly derived from lipolysis-derived FAs/FAs re-esterification (59%) and dietary fat (15%) [22]. Additionally, dietary sugars containing fructose, primarily metabolized in the liver, contribute to the marked increase in DNL in NAFLD patients [23]. In agreement, our work shows that the upregulation of the DNL pathway correlates with sucrose ingestion, resulting in the accumulation of lipid droplets and exacerbated histological severity induced by the HF diet. Other reports have suggested an increased toxicity of SFAs due to their reduced capacity for TG esterification. Conversely, MUFAs were found to be easily incorporated into TG-lipid droplets, thereby protecting against apoptosis and inducing autophagy in vitro and in vivo [24,25]. Indeed, we found that the augmented expression of elongases and desaturases was correlated with the increased incorporation of MUFAs into TGs in all tested diets. Therefore, our results suggest that TGs synthesis is a protective response against the negative effects of SFAs in NAFL.

Hypercaloric diets have been further linked to phospholipid dysregulation in the hepatocytes of NAFLD patients [26]. Gonçalves et al. showed that mitochondria from HF-diet-fed rats exhibit a reduced PC/PE ratio and decreased CL content [27]. Similarly, we observed a reduction in the PC/PE ratio in mice fed a HS diet, but in contrast to their study, we found elevated CL levels in the presence of the HF and/or HS diets. Our data suggest an imbalance in the content of mitochondrial phospholipids that are responsible for the proper structure and fluidity of the mitochondrial membrane in animals fed HS alone or in combination with HF [14]. Interestingly, in vitro studies using liposomes have confirmed that in the presence of increased PE levels, the phospholipid bilayer could maintain its fluidity through a mechanism of augmented membrane viscosity [28]. Therefore, the increased PE content indicates a possible regulatory mechanism of mitochondrial membrane properties in hepatocytes during early NAFLD. Mitochondria play a critical role in the regulation of hepatic lipid metabolism through mt-FAO and OXPHOS. Previous studies have demonstrated that in NAFL, FAs accumulation favors mitochondrial remodeling and mt-FAO enhancement, which in turn promotes mitochondrial respiration [29,30]. Nevertheless, it has been suggested that prolonged inflow of FAs may lead to proton leakage and ROS formation that could ultimately deteriorate mitochondrial OXPHOS, which is proposed to be a key event in NAFLD progression [31,32]. In contrast, our work reports no changes in the mt-FAO pathway. Although we observed reduced levels of OXPHOS subunits and decreased activities of respiratory chain complexes, mitochondrial respiration was sustained in the ADP-stimulated state in animals fed either the HF or HS diet. Importantly, the combined HFHS diet was shown to be more detrimental to mitochondrial respiration than the HF or HS diet. Interestingly, we found elevated levels of CL enriched in MUFAs in all experimental groups but observed a decrease in PUFAs (mostly 18:2, 20:2, 20:4 and 22:6n3) in the HFHS-fed animals. A similar decrease in 18:2 availability and/or defective CL remodeling has been associated with decreased mitochondrial oxygen consumption and diminished OXPHOS complex activities [33,34]. As a signature phospholipid of the IMM, CL is involved in the stabilization of mitochondrial enzymes, particularly in the proper assembly of OXPHOS supercomplexes, and in the maintenance of proper mitochondrial respiration capacity [35]. In line with these observations, our data indicate that an increased CL level may operate as a mitochondrial compensatory mechanism under FA overload. However, we hypothesized that the HFHS-diet-induced changes in the unsaturated acyl chains of CL and in the PC/PE ratio have an impact on mitochondrial structure that could be associated with a reduction in mitochondrial ETC capacity.

Mitochondrial oxidative stress and oxidative damage are the main causes of mitochondrial dysfunction in NAFLD [31,36]. After a detailed characterization of hepatic mitochondrial and cytosolic fractions, we demonstrate that 16 weeks of feeding hypercaloric diets does not induce changes in mitochondrial ROS production or in mitochondrial antioxidant enzyme activities, supporting the results of Einer et al. [14]. Moreover, the absence of mitochondrial oxidative stress is in accordance with the lack of signs of mitochondrial oxidative damage in lipids and proteins. Given that CL deficiency is correlated with increased mitochondrial ROS production [37], the increased levels of this phospholipid found in our study reinforces the finding of reduced mitochondrial oxidative stress in early NAFLD. Although mitochondria are considered the major site of cellular ROS production under pathological conditions, it has been proposed that the ER and peroxisomes may also contribute to ROS generation. In fact, peroxisomes contain several enzymes that produce ROS as part of their catalytic role in metabolic pathways, including FAO [38]. In our study, an assessment of peroxisomal abundance markers and peroxisomal-FAO-related pathway points towards an extensive upregulation of peroxisomal proliferation and peroxisomal-dependent FA metabolism, which is in accordance with previous findings in mouse fatty liver [39,40]. An elegant study by Elsner et al. demonstrated that increased levels of nonesterified FAs induced peroxisomal FAO rather than mt-FAO with the generation of elevated levels of peroxisomal ROS. By easily crossing the peroxisomal membrane, these species may induce the oxidative damage responsible for lipotoxicity in β-pancreatic cells [41]. Likewise, our results suggest that a higher number of peroxisomes combined with the described peroxisomal-mediated induction of FAO activity, in an attempt to reduce lipid accumulation, contributes to ROS production that may overwhelm cytosolic antioxidant enzyme activities. Indeed, we revealed that augmented hepatic lipid peroxide-derived aldehydes contribute to liver oxidative damage in NAFL.

Interestingly, increased peroxisomal ROS production and associated oxidative damage have been linked to a loss of autophagy and the consequent accumulation of functionally compromised peroxisomes [42]. The PI3K/AKT/mTOR nutrient-sensing pathway seems to be intimately involved in the regulation of autophagy. Autophagy appears to protect hepatocytes by removing excess lipid droplets, misfolded proteins and damaged organelles [43], but accumulating evidence has reported autophagy inhibition through the activation of the AKT/mTOR pathway in murine dietary models [44] and in NAFLD patients [45]. Similarly, our data also show autophagic pathway impairment and additionally demonstrate that this effect is diet-type dependent. We found decreased levels of autophagic-related proteins, such as Atg4, the Atg12-Atg5-Atg16 complex and beclin-1, mostly in the HS- and HFHS-diet-fed mice, which have been shown to be responsible for impaired autophagosome formation [44,46]. On the other hand, we also found that compared with the CHOW diet, the HF diet decreased cathepsin B, L and D levels, which may lead to autophagy inhibition by impairing autophagosomal acidification [47,48]. Thus, we propose that HF intake primarily mediates a failed autophagic response dependent on reduced lysosomal enzyme activities and consequent defective autophagosome acidification rather than impaired autophagosome formation. The loss of lysosome acidification capacity is associated with impaired autophagosome degradation and disrupted autophagic flux [49,50]. In our study, the increased levels of SQSTM1/NBR1 and LC3-II observed in the HF- and HFHS-diet-fed mice further support autophagosome accumulation and an autophagic flux blockade induced by HF that confers cytotoxicity.

In conclusion, our study shows that excessive fat and/or sucrose intake exacerbates hepatic steatosis via the accumulation of MUFAs, which protects hepatocytes from SFAs-induced lipotoxicity. Moreover, mitochondrial phospholipid remodeling is mainly associated with increased CL content accompanied by reduced mitochondrial ROS production and mitochondrial oxidative damage. Our work reveals that an increased peroxisomal abundance together with peroxisomal FAO appears to contribute to hepatic oxidative damage and proposes that compromised peroxisomes induce hepatic damage in the early stage of NAFLD development. Moreover, we showed that HS and HF intake compromise the autophagic response at different steps of the degradation process. In particular, fat intake further contributes to the accumulation of damaged cellular components due to compromised autophagic flux. Consequently, the accumulation of malfunctioning organelles may plausibly induce hepatic lipotoxic injuries and NAFL progression to more severe stages of NAFLD.

## Supplementary Materials

The following are available online at https://www.mdpi.com/2076-3921/9/10/995/s1.

## Abbreviations

ΔΨm, mitochondrial membrane potential; ABCD2, ATP-binding cassette subfamily D member 2; ACACA, acetyl-CoA carboxylase 1; ACLY, ATP-citrate synthase; ACOT, acyl-coenzyme A thioesterase; AGPAT9, glycerol-3-phosphate acyltransferase 3; ALT, alanine aminotransferase; AST, aspartate aminotransferase; ATG, autophagy-related protein; BECN1, beclin-1; BHT, butylated hydroxytoluene; CEs, cholesteryl esters; CHOW, standard chow; CL, cardiolipin; DNL, de novo lipogenesis; DNP, dinitrophenyl; DNPH, 2,4-dinitrophenylhydrazine; ELOVL, elongation of very-long-chain fatty acids protein; ETC, electron transport chain; ER, endoplasmic reticulum; FADS, fatty acid desaturase; FAO, fatty acid oxidation; FAs, fatty acids; FASN, fatty acid synthase; GC-MS, gas chromatography-mass spectrometry; GLXR, glutaredoxin; GPX, glutathione peroxidase; GR, glutathione reductase; HF, high-fat; HFHS, high-fat plus high-sucrose; HS, high-sucrose; IMM, inner mitochondrial membrane; LIPE, hormone-sensitive lipase; MDA, malondialdehyde; MGLL, monoglyceride lipase; MPO, myeloperoxidase; mPTP, mitochondrial permeability transition pore; MS, mass spectrometry; MT1, metallothionein-1; mt-FAO, mitochondrial fatty acid oxidation; MUFAs, monounsaturated fatty acids; NAFL, nonalcoholic fatty liver; NAFLD, nonalcoholic fatty liver disease; NAS, NAFLD activity score; NASH, nonalcoholic steatohepatitis; NBR1, autophagy cargo receptor; OXPHOS, oxidative phosphorylation; PC, phosphatidylcholine; PE, phosphatidylethanolamine; PUFAs, polyunsaturated fatty acids; RCR, respiratory control ratio; ROS, reactive oxygen species; SCD-1, acyl-CoA desaturase 1; SEPP1, selenoprotein P; SOD, superoxide dismutase; SFAs, saturated fatty acids; SQST1M, sequestosome-1; TBA, thiobarbituric acid; TGs, triacylglycerols; TLC, thin-layer chromatography; USFAs, unsaturated fatty acids.
