# Supplementary material for: Western Diet Causes Obesity-Induced Nonalcoholic Fatty Liver Disease Development by Differentially Compromising the Autophagic Response
Source: Antioxidants (Basel). 2020 Oct 15;9(10):995. doi: 10.3390/antiox9100995 (PMC7602470; doi:10.3390/antiox9100995)
Supplement: Supplementary file 1 [file antioxidants-09-00995-s001.pdf]

Supplemental Material

Western diet composition causes obesity-induced NAFLD development by differentially compromising the autophagic response

Ines C. M. Simoes[1], Agnieszka Karkucinska-Wieckowska[2], Justyna Janikiewicz[1], Sylwia Szymanska[2], Maciej Pronicki[2], Pawel Dobrzyn[1], Michałl Dabrowski[1], Agnieszka Dobrzyn[1], Paulo J. Oliveira[3], Hans Zischka[4,5], Yaiza Potes[1#*], Mariusz R. Wieckowski[1#*]

Supplemental Material

- Supplemental Methods
- Supplemental Figures
- References

Supplemental Methods

Liver histology. For hematoxylin and eosin (H&E), Masson trichrome staining and other special stains, livers were fixed in 10% phosphate-buffered formalin for 24–48 h at room temperature. Fixed livers were trimmed to the appropriate size and shape and placed in embedding cassettes. The paraffin embedding schedule was as follows (16 h total): 70% ethanol, two changes, 1 h each; 80% ethanol, one change, 1 h; 95% ethanol, one change, 1 h; 100% ethanol, three changes, 1.5 h each; xylene, three changes, 1.5 h each; and paraffin wax (58–60 °C), two changes, 2 h each to embed tissues into paraffin blocks. For Oil Red staining, livers were placed in tissue embedding medium (matrix) and frozen in isopentane cooled by liquid nitrogen.

a) For H&E and Masson trichrome staining, paraffin blocks were cut into 3 µm thick slices and mounted on SuperFrost microscope slides (Gerhard Menzel GMBH, Braunschweig, D-

38116 Germany). After stepwise deparaffinization and rehydration, the slides were stained with H&E according to standard protocols [1]. The standard protocol of the manufacturer for Masson trichrome staining was followed with the use of Bouin's solution (Sigma-Aldrich Sp. z. o. o., 61-626 Poznan, Poland; catalog no. HT10132), Weigert's iron hematoxylin solution (Sigma-Aldrich Sp. z o.o., 61-626 Poznan, Poland; catalog no. HT1079-1SET) and a Trichrome Stain (Masson) Kit (Sigma-Aldrich Sp. z o.o., 61-626 Poznan, Poland; catalog no. HT15-1KT) [1]. Masson trichrome staining is intended for use in the study of connective tissue, collagen fibers (blue), muscle (red) and nuclei (black) [2].

b) Other specific stains were performed on the BenchMark Special Stains platform using a ready-to-use Ventana staining kit (Supplemental Table 2) after automatically executed deparaffinization and rehydration.

c) For Oil Red staining, after freezing tissue embedding medium, the livers were cut into 8 µm thick slices and mounted on SuperFrost Plus microscope slides (Gerhard Menzel GMBH, Braunschweig, D-38116 Germany). Neutral lipid accumulation in livers from CHOW, HF, HS and HFHS diet-fed mice was visualized with Oil Red staining [1]. Staining with oil-soluble dyes is based on the greater solubility of the dye in the lipoid substances than in typical hydroalcoholic dye solvents [3].

d) Immunohistochemistry analysis of the CD3, CD4 and CD68 inflammation markers was performed on formalin-fixed paraffin-embedded (FFPE) tissue samples. The expression of CD3 protein was detected using rabbit polyclonal antibody (A0452, Agilent, Santa Clara, USA); CD4 (orb4830, Biorbyt, St Louis, USA) and CD68 (GTX37743, GeneTex, Alton Pkwy Irvine, USA) expression was also detected. Antigen retrieval was performed using Target Retrieval Solution at low pH (DAKO, Glostrup, Denmark) for 30 minutes at 99.5 °C.

e) All above mentioned stains were scanned in a Hamamatsu NanoZoomer 2.0 RS scanner (Hamamatsu Photonics, Hamamatsu, Japan) at the original magnification of 40×.

f) For the NAFLD Activity Score (NAS): a histopathological evaluation of steatosis (grade 0-3), ballooning (grade 0-2) and lobular inflammation (grade 0-3) was performed by a pathologist from The Children's Memorial Health Institute. The system of evaluation is described by Kleiner et al. [4]. The final score is well defined by the unweighted sum of the scores obtained for steatosis, lobular inflammation, hepatocyte ballooning and fibrosis.

Mass spectrometry analysis. Liquid chromatography-MS3 spectrometry (LC-MS/MS) was carried out at the Thermo Fisher Center for Multiplexed Proteomics (Department of Cell Biology, Harvard Medical School, Cambridge, MA, USA). Peptide fractions were analyzed using an LC-MS3 data collection strategy on an Orbitrap Fusion mass spectrometer (Thermo Fisher Scientific Inc., Waltham, MA, USA).

a) Quantitative mass spectrometry analysis. All solutions are reported as final concentrations. Lysis buffer (8 M urea, 1% SDS, 50 mM Tris (pH 8.5), protease and phosphatase inhibitors from Roche, Basel, Switzerland) was added to the liver samples to achieve a protein concentration between 2-8 mg/mL. A micro-BCA assay (Pierce Biotechnology Inc., Rockford, IL, USA) was used to measure the final protein concentration in the tissue lysate. The proteins were reduced and alkylated as previously described. Then, the proteins were precipitated using methanol/chloroform. In brief, four volumes of methanol were added to the tissue lysate, followed by one volume of chloroform and finally three volumes of water. The mixture was vortexed and centrifuged to separate the chloroform phase from the aqueous phase. The precipitated protein was washed with one volume of ice-cold methanol. The washed precipitated protein was allowed to air dry. The precipitated protein was then resuspended in 4 M urea and 50 mM Tris (pH 8.5). The proteins were first digested with LysC (1:50; enzyme:protein) for 12 h at 25 °C. The LysC digestion was diluted to 1 M urea and 50 mM Tris (pH 8.5) and then digested with trypsin (1:100; enzyme:protein) for another 8 h at 25 °C. Next, the peptides were desalted using C18 solid phase extraction cartridges as

previously described, and the dried peptides were resuspended in 200 mM EPPS (pH 8.0). Peptide quantification was performed using a micro-BCA assay (Pierce). The same amount of peptide from each condition was labeled with tandem mass tag (TMT) reagent (1:4; peptide: TMT label, Pierce). The 10-plex labeling reactions were performed for 2 h at 25 °C. Modification of tyrosine residues with TMT was reversed by the addition of 5% hydroxyl amine for 15 minutes at 25 °C. The reaction was quenched with 0.5% TFA, and samples were combined at a 1:1:1:1:1:1:1:1:1:1 ratio for 10-plex experiments. The combined samples were desalted and fractionated offline into 24 fractions as previously described [7].

b) LC-MS/MS. Twelve of the 24 peptide fractions from the basic reversed-phase step (every other fraction) were analyzed with an LC-MS3 data collection strategy [8] on an Orbitrap Fusion mass spectrometer (Thermo Fisher Scientific Inc., Waltham, MA, USA) equipped with a Proxeon Easy nLC 1000 for online sample handling and peptide separations. Approximately 5 µg of peptide resuspended in 5% formic acid + 5% acetonitrile was loaded onto a 100 µm inner diameter fused-silica micro capillary with a needle tip pulled to an internal diameter less than 5 µm. The column was packed in-house to a length of 35 cm with a C18 reversed-phase resin (GP118 resin, 1.8 µm, 120 Å, Sepax Technologies Inc., Newark, DE, USA). The peptides were separated using a 120-minute linear gradient from 3% to 25% buffer B (100% ACN + 0.125% formic acid) equilibrated with buffer A (3% ACN + 0.125% formic acid) at a flow rate of 600 nL/minute across the column. The scan sequence for the Fusion Orbitrap began with an MS1 spectrum (Orbitrap analysis; resolution, 120,000; 400−1400 m/z scan range; AGC target, 2 × 105; maximum injection time, 100 ms; dynamic exclusion, 75 seconds). 'Top speed' (2 seconds) was selected for MS2 analysis, which consisted of CID (quadrupole isolation set at 0.5 Da and ion trap analysis; AGC, 4 × 103; NCE, 35; maximum injection time, 150 ms). The top ten precursors from each MS2 scan were selected for MS3 analysis (synchronous precursor selection), in which precursors were

fragmented by HCD prior to Orbitrap analysis (NCE, 55; max AGC, 5 × 104; maximum injection time, 150 ms; isolation window, 2.5 Da; resolution, 60,000).

c) LC-MS3 data analysis. A suite of in-house software tools was used for RAW file processing and controlling peptide and protein level false discovery rates, assembling proteins from peptides, and protein quantification from peptides as previously described. MS/MS spectra were searched against a UniProt mouse database (2014) with both the forward and reverse sequences. The database search criteria were as follows: tryptic with two missed cleavages, a precursor mass tolerance of 50 ppm, fragment ion mass tolerance of 1.0 Da, static alkylation of cysteine (57.02146 Da), static TMT labeling of lysine residues and N-termini of peptides (229.162932 Da), and variable oxidation of methionine (15.99491 Da). TMT reporter ion intensities were measured using a 0.003 Da window around the theoretical m/z for each reporter ion in the MS3 scan for 10-plex data (0.03 for 6-plex data). Peptide spectral matches with poor quality MS3 spectra were excluded from quantitation (<200 summed signal-to-noise across 10 channels, <100 for 6-plex and <0.5 precursor isolation specificity).

Mass spectrometry data analysis. Protein data (ProteinQuant) from two independent quantification experiments were uploaded into the mysql database and merged using Protein ID (SwissProt/UniProt ID). This resulted in a table with 7365 distinct protein IDs. Each quantification run consisted of several plexes (groups of 10 samples analyzed together), with each plex including the same reference sample. Separately, the data for each plex were normalized by dividing the quantification result for each protein (protein ID) in a particular sample by the (nonzero) quantification result for the same protein in the reference sample. If the quantification result for a protein in the reference sample of a given plex had a value of zero or null, then the normalization result for this protein in this plex was set to null. The dataset was split into two parts: 4677 rows that contained no null values and 2689 rows that

contained one or more null values. The data with no null values, consisting of 4677 rows x 23 columns, were clustered with the number of significant clusters determined by the gap statistics. The mean difference data (between every group and the control group) were loaded into KeggAnim for visualization of specific pathways [9]. The expression of selected groups of proteins was visualized by heatmaps.

Supplemental Table 1. Protein, fiber, N-free extracts, starch, sugar, fat (%) and cholesterol (mg/kg) composition of the CHOW and HF diets, both purchased from Ssniff.

**Composition**	**Standard chow diet** (Ssniff V1534)	**High-fat diet** (Ssniff E15126)
Protein (%)	19.00	20.70
Fiber (%)	4.90	5.00
N-free extracts (%)	54.10	34.30
Starch (%)	36.50	17.20
Sugar (%)	4.70	16.30
Fat (%)	3.30	30.00
C12:0	-	0.03
C14:0	0.01	0.99
C16:0	0.47	7.49
C16:1	0.01	0.74
C17:0	-	0.36
C18:0	0.08	5.29
C18:1	0.62	10.98
C18:2	1.80	0.75
C18:3	0.23	0.13
C20:0	0.01	0.03
C20:1	0.02	0.01
C20:4	-	0.07
Cholesterol (mg/kg)	-	284.00
Metabolizable energy (MJ/kg)	12.80	20.10

Supplemental Table 2. List of other special staining kits used with the catalog number, method and expected results of each stain.

Staining kit name	Catalog number	Methods	Observed results
PAS staining kit (periodic acid + Schiff reagent)	860-014	The PAS staining kit is intended for use as a qualitative histologic stain to demonstrate the presence of glycogen.	Glycogen, glycolipids, and glycoprotein – purple;<br>nuclei - blue
Diastase kit	860-004	The diastase kit, in conjunction with the PAS Staining Kit, is intended for use as a digestion reagent to aid in the analysis of glycogen.	Only glycogen is sensitive to diastase digestion.
Reticulum II	860-024	The reticulum II staining kit is intended for use as a qualitative histologic stain to label reticular fibers.	Reticular fibers stain black against a pink to red background.

Supplemental Table 3. Histopathological evaluation of the NAS. Steatosis grade: grade 0 = < 5%; grade 1 = 5–33%; grade 2 = 34–66%; and grade 3 = > 66%. Ballooning grade: grade 0 = absent; grade 1 = few ballooned hepatocytes; and grade 2 = many ballooned hepatocytes. Lobular inflammation grade: grade 0 = absent; grade 1 = up to two foci per field of view (200x magnification); grade 2 = two to four foci per field of view; and grade 3 = more than four foci per field of view (note: lipogranulomas are included in the category of inflammation). Fibrosis grade: S0 = no fibrosis; S1a = zone 3, perisinusoidal fibrosis; S1b = zone 3, perisinusoidal fibrosis, can be detected with H&E; S1c = only periportal/portal fibrosis; S2 = zone 3, plus portal/periportal fibrosis; S3 = zone 3, plus portal/periportal fibrosis with bridging fibrosis; and S4 = cirrhosis. [&]NAS score: sum of the steatosis, hepatocyte ballooning, lobular inflammation and fibrosis grades. HF, high-fat; HFHS, high-fat plus high-sucrose; HS, high-sucrose.

**NAFLD Activity Score (NAS)**					**NAS value[&]**
**Groups**	**Steatosis**	**Ballooning**	**Inflammation**	**Fibrosis**	
CHOW	grade 0	grade 0	grade 0	S 0	0
HF	grade 3	grade 2	grade 0	S 0	5
HS	grade 3	grade 2	grade 0	S 0	5
HFHS	grade 3	grade 2	grade 0	S 1a	6

Supplemental Figures

Supplemental Figure 1. HF and HFHS diets induce simple steatosis with alterations in the normal architecture of hepatocytes. (A) Hepatic glycogen levels obtained from three independent images (per animal) from the experiments shown in (B) (N=4). **(B)** Representative images of paraffin-embedded liver sections with PAS and PAS diastase staining. Scale bar, 250 µm; magnification, 10x. **(C)** Hepatic reticular fiber levels obtained

from three independent images (per animal) from the experiments shown in (D) (*N*=4). **(D)** Representative images of paraffin-embedded liver sections with reticulum staining. Scale bar, 250 µm; magnification, 10x. All data are expressed as the mean ± SEM. (*) vs. the CHOW diet ($P < 0.05$); (**) vs. the CHOW diet ($P < 0.01$); (***) vs. the CHOW diet ($P < 0.001$); *P* values were determined using one-way ANOVA followed by Bonferroni *post hoc* test. HF, high-fat; HFHS, high-fat plus high-sucrose; HS, high-sucrose; PAS, periodic acid Schiff.

Supplemental Figure 2. HFHS diet induces a decrease in PUFAs in mitochondrial PC but an increase in PUFAs in mitochondrial PE. (A) Content of SFAs, MUFAs and PUFAs in the PC of isolated hepatic mitochondria and detailed analysis of FA acyl chains in PC (*N*=4). **(B)** Content of SFAs, MUFAs and PUFAs in the PE of isolated hepatic mitochondria and detailed analysis of FA acyl chains in PE (*N*=4). All data are expressed as the mean ± SEM. (*) vs. the CHOW diet and (§) vs. the HF diet ($P < 0.05$); (**) vs. the CHOW diet and (§§) vs. the HF diet ($P < 0.01$); (***) vs. the CHOW diet ($P < 0.001$); *P* values were determined using one-way ANOVA followed by Bonferroni *post hoc* test or Kruskal-Wallis test followed by Dunn *post hoc* test. HF, high-fat; HFHS, high-fat plus high-sucrose; HS, high-sucrose; PC, phosphatidylcholine; PE, phosphatidylethanolamine; PUFA, polyunsaturated fatty acid; SFA, saturated fatty acid; USFA, unsaturated fatty acid.

Supplemental Figure 3. HF, HS and HFHS diets diminish the levels of OXPHOS subunits and assembly factors. (A) Proteomic analysis of the levels of Complex I subunits and assembly factors (*N*=4). **(B)** Complex II subunit levels (*N*=4). **(C)** Complex III subunit and assembly factor levels (*N*=4). **(D)** Complex IV subunit and assembly factor levels (*N*=4). **(E)** Complex V subunit and assembly factor levels (*N*=4). Blue represents decreased levels,

and red represents increased levels. HF, high-fat; HFHS, high-fat plus high-sucrose; HS, high-sucrose.

SUPPLEMENTARY REFERENCES

1. Szymanska-Debinska, T., et al., *Leigh disease due to SCO2 mutations revealed at extended autopsy.* J Clin Pathol, 2015. **68**(5): p. 397-9.
2. Bancroft, J.D. and C. Layton, *12 - Connective and other mesenchymal tissues with their stains*, in *Bancroft's Theory and Practice of Histological Techniques (Eighth Edition)*, S.K. Suvarna, C. Layton, and J.D. Bancroft, Editors. 2019, Content Repository Only! p. 153-175.
3. Preece, A., *A manual for Histologic Technicians*. 3rd Ed ed. 1972.
4. Kleiner, D.E., et al., *Design and validation of a histological scoring system for nonalcoholic fatty liver disease.* Hepatology, 2005. **41**(6): p. 1313-21.
5. Weekes, M.P., et al., *Quantitative temporal viromics: an approach to investigate host-pathogen interaction.* Cell, 2014. **157**(6): p. 1460-72.
6. McAlister, G.C., et al., *MultiNotch MS3 enables accurate, sensitive, and multiplexed detection of differential expression across cancer cell line proteomes.* Anal Chem, 2014. **86**(14): p. 7150-8.
7. Adler, P., et al., *KEGGanim: pathway animations for high-throughput data.* Bioinformatics, 2008. **24**(4): p. 588-90.
